# Pleasantness and trigeminal sensations as salient dimensions in organizing the semantic and physiological spaces of odors

**DOI:** 10.1038/s41598-018-26510-5

**Published:** 2018-05-31

**Authors:** C. C. Licon, C. Manesse, M. Dantec, A. Fournel, M. Bensafi

**Affiliations:** Lyon Neuroscience Research Center, CNRS UMR5292, INSERM U1028, Université Claude Bernard Lyon 1, 50 avenue Tony Garnier, 69366 Lyon Cedex 07, France

## Abstract

A major issue in human olfaction research is to characterize the main dimensions that organize the space of odors. The present study examines this question and shows that, beside pleasantness, trigeminal sensations, and particularly irritation, play an important role. These results were consistent along two different spaces constructed using semantic description and physiological responses to 105 odorants, smelled and described by human participants. Taken together, these findings suggest that salient trigeminal features, in conjunction with pleasantness, are involved in detecting relevant emotional stimuli, and modify the way organisms categorize smells. These results shed light on the importance of trigeminal sensitivity in the well-established defensive function of olfaction.

## Introduction

From a biological point of view, olfaction is a prominent sense for humans; and, besides its social role, one of its main functions is to prevent danger, as seen in approach/avoidance behavior^1^. Should I avoid or approach the stimulus? Should I eat it or not? The psychological or perceptual dimensions that sustain these behaviors are called hedonic valence or pleasantness (for the approach/avoidance axis) and edibility (for the eat/not eat axis)^1^. Nevertheless, what is known as “odor” actually comprises both olfactory and trigeminal sensory attributes^2^. These two systems enable the construction of a whole chemosensory experience. The perceptual attributes of smells enable the subject to like/dislike and/or recognize olfactory sources. The trigeminal system has complementary functions, such as detection of irritant or poisonous chemicals so as to be able to avoid them. In general, the trigeminal system provides information regarding sensations such as warmth or coolness, and pain or irritation. These sensations are produced by almost all odorant molecules, depending on concentration levels^3^. The trigeminal system also enables detection and recognition of appetitive environmental sources (e.g., via the cool sensation of a minty candy).

One important question in the field is to understand how perceptual (i.e., pleasantness, edibility) and trigeminal attributes influence olfactory processing. Human olfactory responses appear on both verbal and psychophysiological levels, and very often on both together^4^. However, the literature in the scientific and clinical fields has mainly focused on the influence of perceptual dimensions (such as pleasantness^5–8^) on both verbal and physiological responses to odors, neglecting the trigeminal attributes of smells.

Previous studies of verbal response focused largely on both pleasantness and edibility: the pleasantness dimension was given a strong weight, as the first attribute that humans use to categorize smells^9–12^. Past investigations also suggested that the edibility of odor sources is also an important factor that drives the organization of the perceptual space of odors; yet it was very often ranked below pleasantness^1,12,13^. In line with this hypothesis, neuroscientific studies revealed that both approach/avoidance behavior in animals and odorant toxicity are predicted by overall neural activity in the olfactory system, although approach/avoidance behavior was predicted more strongly than odorant toxicity^1^.

Studies of psychophysiological response showed that skin conductance variations were modulated by odor quality^14^ and pleasantness^15^. Skin temperature is also modulated by odor type and perceived intensity^16^. Finally, odor pleasantness influences respiration and sniffing^4,17,18^.

As mentioned above, unlike perceptual dimensions (intensity, pleasantness and edibility), the impact of the trigeminal dimensions of odorants on the organization of both the semantic and physiological spaces of odors has been barely studied. Therefore, the aim of the present study was to characterize the respective weights of a series of perceptual dimensions (pleasantness and edibility, but also familiarity and intensity) and trigeminal dimensions (coolness, warmth, irritation and pain) at two levels of olfactory processing: semantic and physiological.

To this aim, a sample of individuals was tested in a two-session study. In the first session, participants were asked to smell 105 different odorants and to rate them along emotional dimensions, while their psychophysiological responses were recorded. In the second session (one to three days later), the same volunteers were asked to smell the same 105 odorants and rate them according to 24 predefined olfactory qualities (with no reference to the above 8 perceptual and trigeminal dimensions of interest). During this second session, odorants were also rated along the 8 dimensions of interest: pleasantness, intensity, familiarity, edibility, coolness, warmth, irritation and pain. The semantic space of odors was constructed using the 24 qualities but excluding the perceptual and trigeminal dimensions of interest, and the physiological space of odors was thus built on the basis of autonomic nervous system (ANS) responses.

## Results

### Reproducibility from session one to session two

**Figure 1:**
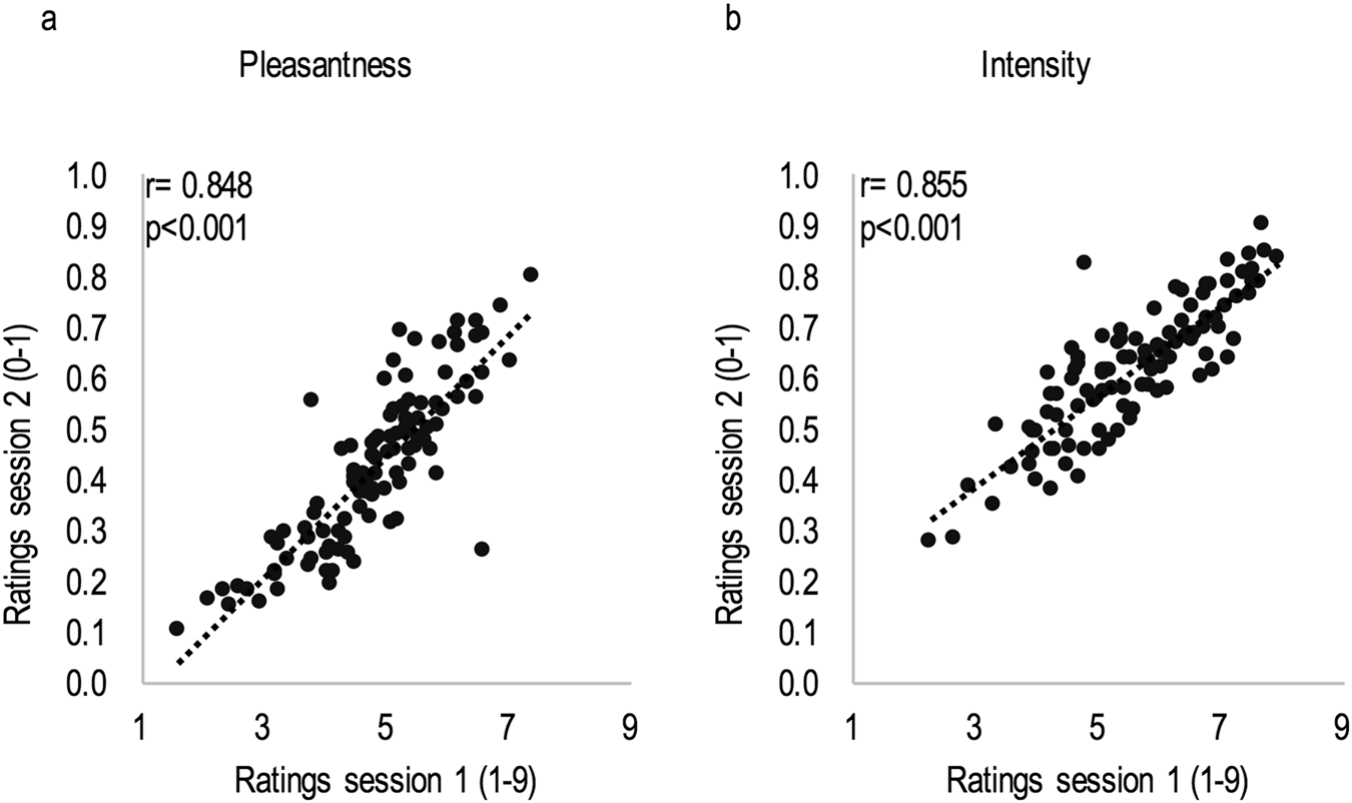
Reproducibility analysis. Correlation factor (r) between session one and session two for (**a**) pleasantness ratings and (**b**) intensity ratings of the 105 odorants.

To test the consistency of psychophysical evaluations between sessions, participants were asked to rate pleasantness and intensity in session one and in session two. All odorant compounds were presented at the same concentration and random order in both sessions. Results showed a significant positive correlation for pleasantness ratings (r = 0.848, p < 0.001) (Fig. 1a) and for intensity ratings (r = 0.855, p < 0.001) (Fig. 1b). Both the pleasantness and the intensity ratings of the 105 odorants were homogeneously distributed along the range of the scales used, although for intensity ratings no stimuli were rated less than two on average.

### Building the semantic space of odors

To build the semantic space without any direct influence of the perceptual (pleasantness, intensity, familiarity and edibility) and trigeminal dimensions (irritation, pain, warmth and coolness), we used descriptor data collected from the participants in session two, where they were asked to rate whether and how strongly (from 0 to one on a continuous scale) each of 24 predefined semantic descriptors applied to the stimuli (floral rose, floral others, fruity citrus, fruity others, oily, vegetal, herbaceous, earthy, woody, smoky, cooked food, spicy, nutty, medicinal/minty/camphor, ethereal/disinfectant, alliaceous, chemical, fermentation, lactic, animal, human, decay, anisic and sweet/balsamic; see Methods). The most commonly used descriptors were “chemical”, “woody” and “herbaceous”, and the least frequently “body odors”, “citrus” and “floral rose”. Principal component analysis revealed eight significant components with eigenvalue >1, explaining 68.94% of the variance. Additional analyses (bootstrap eigenvalues, parallel analysis) gave four significant components, explaining 47% of the variance. The first component showed a positive significant correlation with the descriptors “decay” (r = 0.873, p < 0.001), “fermentation” (r = 0.752, p < 0.001) “animal” (r = 0.749, p < 0.001), “body odors” (r = 0.719, p < 0.001), “lactic” (r = 0.683, p < 0.010) and “alliaceous” (r = 0.623, p < 0.010), while the second one was negatively correlated with “nutty” (r = −0.713, p < 0.010), “cooked food” (r = −0.684, p < 0.001), “woody” (r = −0.540, p < 0.010) and “smoked” (r = −0.506, p < 0.050). Regarding the third component, it was positively correlated with “ethereal” (r = 0.668, p < 0.001) and negatively correlated with “fruity” (r = −0.541, p < 0.010) and “sweet” (r = −0.503, p < 0.050). Finally, the fourth principal component was positively correlated with “sweet” (r = 0.503, p < 0.050) and negatively correlated with “herbaceous” (r = 0.582, p < 0.010), “woody” (r = −0.539, p < 0.010), and “earthy” (r = −0.520, p < 0.010).

### Relating the semantic space to olfactory and trigeminal dimensions

**Figure 2:**
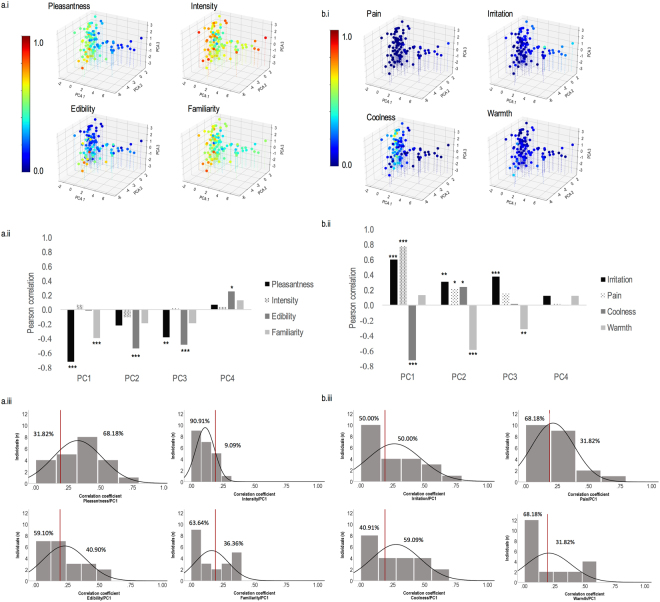
Semantic space of odors. (**a**) (i) Three-dimensional representation of odors in the semantic space as a function of pleasantness, intensity, edibility and familiarity; (ii) correlation coefficients between the first four principal components of the PCA (PC1 to PC4) and each of the four olfactory perceptual dimensions (pleasantness, intensity, edibility, familiarity). (iii) Distribution of individual absolute values of coefficient correlations (r) between the four olfactory perceptual dimensions and the first principal component of the semantic space. (**b**) (i) Three-dimensional representation of odors in the semantic space as a function of pain, irritation, coolness and warmth; (ii) correlation coefficients between the first four principal components of the PCA and each of the 4 trigeminal dimensions of pain, irritation, coolness and warmth). (iii) Distribution of individual absolute values of coefficient correlations (r) between the four trigeminal dimensions and the first principal component of the semantic space. In a.i and b.i, color scale codes for each of the eight perceptual ratings: ranges from 0 (blue) to 1 (red). (*p < 0.05, **p < 0.01, ***p < 0.001).

To understand the meaning of the principal components of the resulting semantic space, we performed correlation analyses between each of the four significant principal components and the dimensions of intensity, pleasantness, familiarity and edibility. Replicating previous studies^1,9,13^, results revealed that pleasantness correlated significantly with the first (r = −0.719, p < 0.001) and third (r = −0.380, p < 0.010) principal components of the semantic space. Familiarity correlated negatively, to a lesser degree, with the first principal component (r = −0.394, p < 0.001). Another result of interest was that edibility correlated significantly with the second (r = −0.540, p < 0.001), third (r = −0.488, p < 0.001) and fourth (r = 0.257, p < 0.050) principal components (Fig. 2ai and aii).

The other four non-significant principal components showed significant correlations with some of the dimensions: PC5 showed significant correlations with familiarity (r = −0.252, p < 0.050), edibility (r = −0.334, p < 0.010), and coolness (r = −0.438, p < 0.001); PC6 was significantly correlated with intensity (r = 0.233, p < 0.050). PC7 did not showed any significant correlation and PC8 showed a significant correlation with intensity (r = 0.245, p < 0.050).

We then considered the 4 trigeminal dimensions, and performed correlation analyses between each of the 4 principal components and the dimensions of irritation, pain, warmth and coolness. Results revealed that pain was the trigeminal dimension most strongly correlated with the first principal component of the semantic space (r = 0.777, p < 0.001) and, to a lesser degree, to the second (r = 0.224, p < 0.050); pain was followed by irritation (r = 0.600, p < 0.001) in the case of the first component; irritation also correlated with the second (r = 0.312, p < 0.010) and third (r = 0.380, p < 0.001) principal components; coolness correlated with the first (r = −0.718, p < 0.001) and second components (r = 0.247, p < 0.050); and finally, warmth correlated with the second (r = −0.588, p < 0.001) and third (r = −0.312, p < 0.010) principal components. No significant correlations were obtained for the fourth principal component (Fig. 2bi and bii).

In a complementary analysis, we asked to which extent perceived intensity explained the above results. To this end, we performed the exact same correlational analyses but used intensity ratings as a co-factor. Results revealed the same pattern for both the olfactory dimensions and the trigeminal dimensions (all significant correlations remained significant after correcting for intensity ratings). A minor change was notable in that correlations between the first principal component on the one hand and pleasantness (r = 0.780) and familiarity (r = 0.600) on the other hand increased.

To further consider individual variance and explore whether the above findings were observed at the level of the individual, the same principal component analyses were performed within each participant. Whereas the group analysis considered the correlation between the principal components of the olfactory space and perceptual dimensions, the individual analysis examined the percentage of individuals for whom the principal components of the olfactory and olfactory/trigeminal dimensions were significantly correlated. For olfactory dimensions, results revealed that the first principal component was correlated significantly with pleasantness, edibility, familiarity and intensity in respectively 68.18%, 40.90%, 36.36% and 9.09% of the participants. For the trigeminal dimensions, significant correlations between the first principal component on the one hand and coolness, irritation, warmth and pain on the other hand were observed in respectively 59.09%, 50.00%, 31.82%, 31.82%. As in the group analysis, the percentage of participants who exhibited significant correlations was lower for the remaining components. For example, for the second principal component, significant correlations were observed with pleasantness, edibility, familiarity and intensity in respectively 50.00%, 40.91%, 9.09% and 0.00%. For the trigeminal dimensions of coolness, irritation, warmth and pain significant correlations were observed in 40.91%, 18.18%, 13.64% and 13.64%. In sum, such analysis taking into account individual variance did not show that correlations between the principal components and the olfactory and trigeminal dimensions were not significantly correlated in all individuals, it highlighted – as the group analysis – that the main principal component (PC1) was correlated significantly with dimensions of pleasantness, edibility, coolness and irritation in a large proportion of individual (at least 40%).

**Figure 3:**
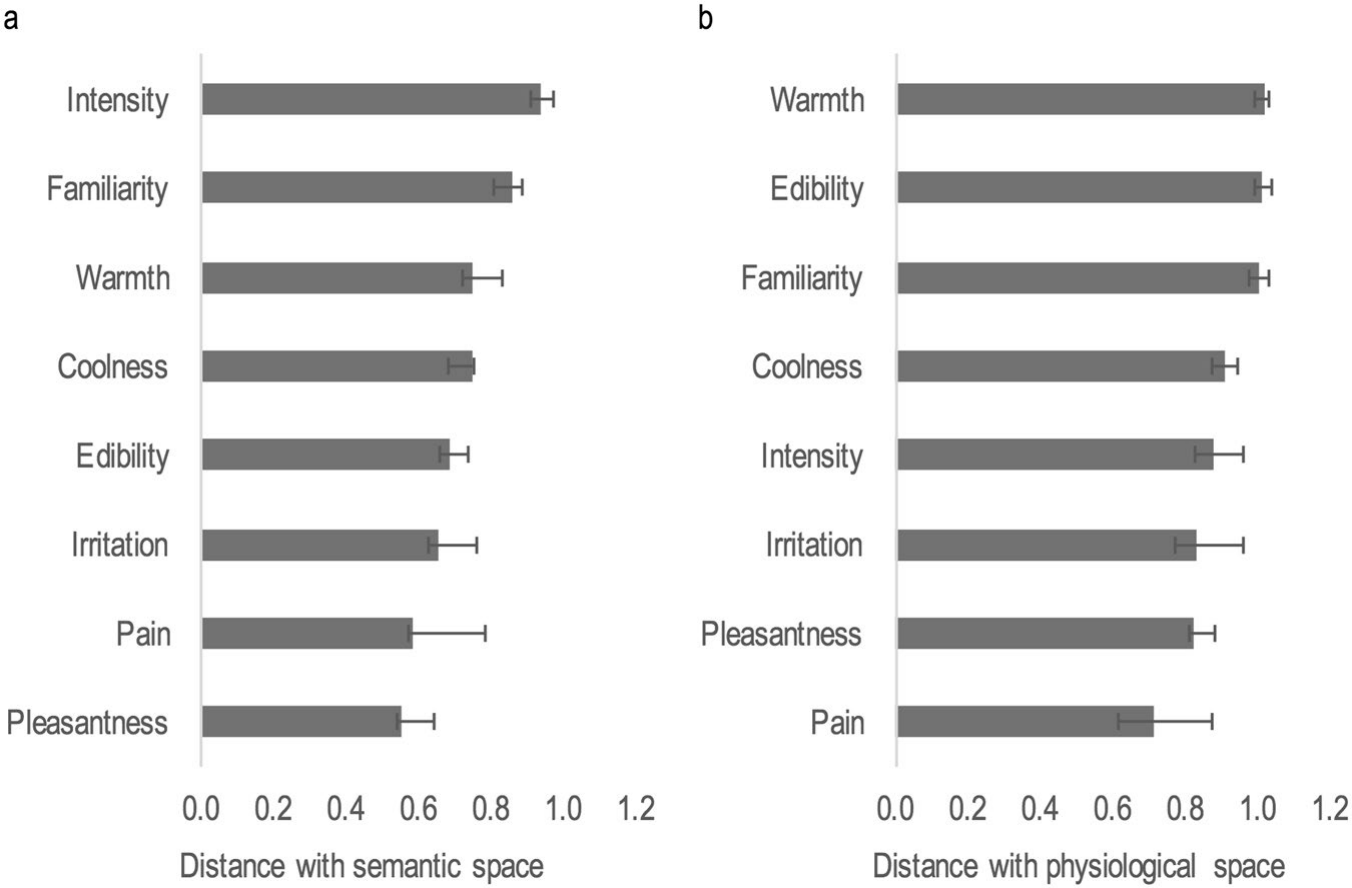
Distance similarity. Comparison of distance between odorants in t﻿﻿he eight perceptual spaces and (**a**) the semantic space and (**b**) the physiological space of odors . Each bar represents the distance similarity index; error bars illustrate standard errors. Dimensions are ordered from most similar to most different.

We then performed a similarity analysis at the level of the group by comparing distances between stimuli in the semantic space on the one hand and each of the 8 perceptual and trigeminal spaces on the other hand. This analysis revealed that the strongest correlations between the semantic space and the one-dimensional perceptual spaces were observed for pleasantness (DS = 0.549, p < 0.01), pain (DS = 0.580, p < 0.01), irritation (DS = 0.654, p < 0.01) and edibility (DS = 0.683, p < 0.01). These were followed by coolness (DS = 0.745, p < 0.01), warmth (DS = 0.749, p < 0.01), familiarity (DS = 0.857, p < 0.01) and intensity (DS = 0.939, p > 0.05) (Fig. 3a).

### Building the physiological space of odors

The physiological space of odors was constructed using each of the 6 physiological parameters recorded in session one (three skin conductance parameters, plus respiration, heart rate and skin temperature; see Methods). Principal component analysis revealed two components with eigenvalue >1, explaining 56.17% of the variance. Additional analyses (bootstrap eigenvalues, parallel analysis) gave only one significant component, explaining 36.17% of the variance. The first component showed significant negative correlations with the 3 skin conductance parameters of rise time (r = −0.931, p < 0.001), latency (r = −0.808, p < 0.001) and amplitude (r = −0.747, p < 0.001), and with respiration rate (r = −0.268, p < 0.010).

### Relating the physiological space to olfactory and trigeminal dimensions

**Figure 4:**
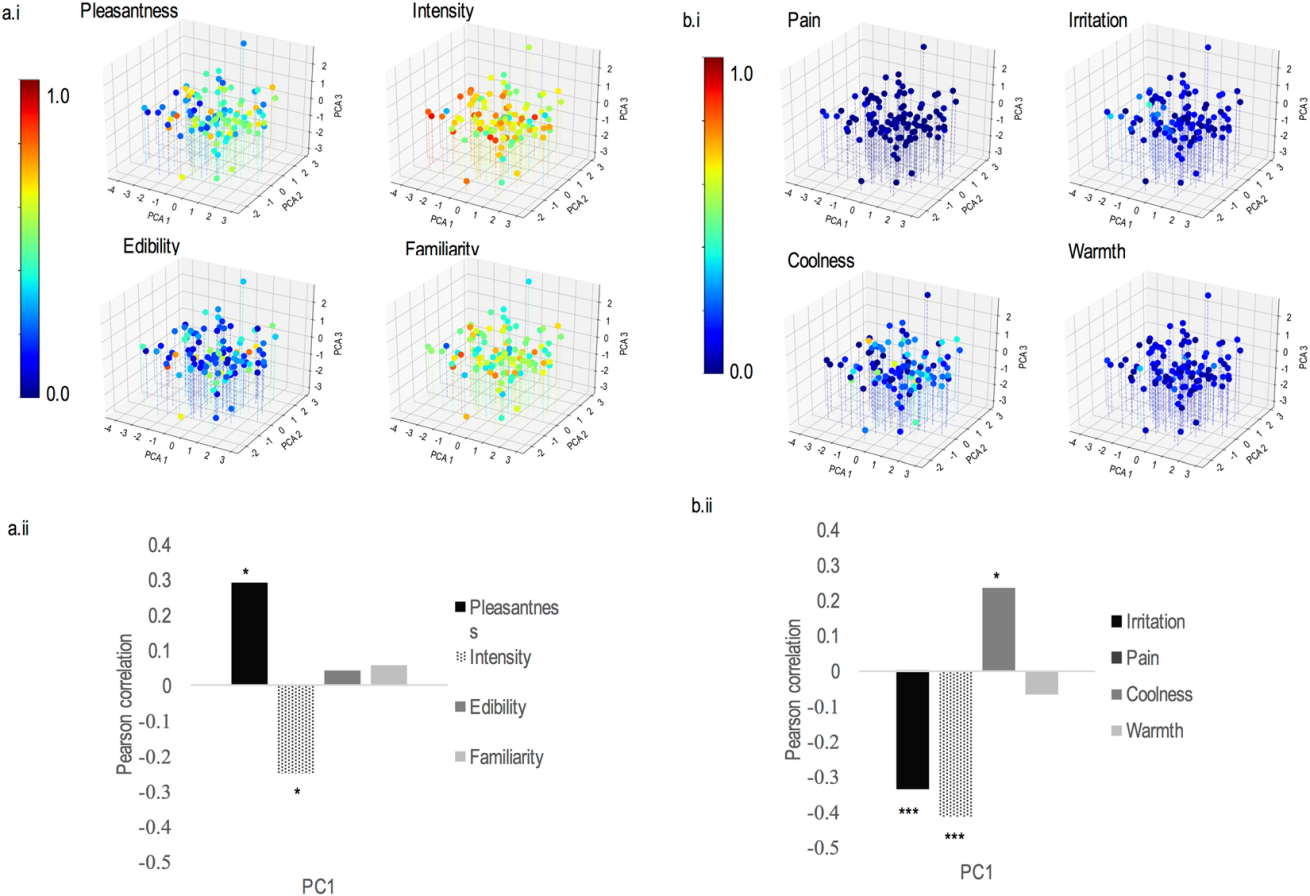
Physiological space of odors. (**a**) (i) Three-dimensional representation of odors in the physiological space as a function of pleasantness, intensity, edibility and familiarity; (ii) correlation coefficients between the first principal component of the PCA (PC1) and each of the four olfactory perceptual dimensions (pleasantness, intensity, edibility, familiarity). (**b**) (i) Three-dimensional representation of odors in the physiological space as a function of pain, irritation, coolness and warmth; (ii) correlation coefficients between the first principal component of the PCA and each of the four trigeminal dimensions (pain, irritation, coolness and warmth). In a.i and b.i, color scale codes for each of the eight perceptual ratings: ranges from 0 (blue) to 1 (red). (*p < 0.05).

Correlation analyses between the first principal component of the physiological space and the four perceptual dimensions (intensity, pleasantness, familiarity and edibility) revealed significant relationships with pleasantness (r = 0.291, p < 0.050) and intensity (r = −0.248, p < 0.050) (Fig. 4ai and aii). Analysis on the trigeminal dimensions revealed significant relationships between the physiological space and the dimensions of pain (r = −0.414, p < 0.001), coolness (r = 0.237 p < 0.050) and irritation (r = −0.335 p < 0.001) (Fig. 4bi,bii).

Like for the semantic space, similarity analysis was also performed for the physiological space and the 8 dimensions of interest. Results revealed that the best correlations between the physiological space and the one-dimensional perceptual spaces were observed for pain (DS = 0.711, p < 0.010), pleasantness (DS = 0.820, p < 0.010) and irritation (DS = 0.828, p < 0.050). These were followed by intensity (DS = 0.877, p < 0.010), coolness (DS = 0.908, p > 0.050), familiarity (DS = 1.001, p > 0.050), edibility (DS = 1.011, p > 0.050) and warmth (DS = 1.021, p > 0.050) (Fig. 3b).

## Discussion

Understanding the organization of the olfactory space is one of the main challenges in the field of human olfaction. Some authors^11,19^ suggest that the human perceptual space of odors may have only a few dimensions, governed by behavioral and ecological constraints, while others suggest that it is multidimensional^13^. To gain new insight on this topic, the present study examined the contributions of perceptual (pleasantness, edibility, familiarity, intensity) and trigeminal dimensions (irritation, pain, coolness and warmth) to the organization of a semantic and a physiological space of odors. Results showed that salient dimensions in both spaces included pleasantness, irritation, coolness and pain. In agreement with previous psychophysiological studies, odor intensity ratings correlated significantly with the physiological space of odors^20^. This difference was, however, ranked fourth, after the dimensions of pleasantness, irritation and pain, and no correlations were observed for intensity in the semantic space. Considering the other dimensions, familiarity was found to be significantly associated with the first principal component of the semantic space of odors, and edibility and warmth with the second and third principal components. None of these dimensions was associated with the physiological space. Taken together, these findings suggest that the organization of the odor space may be hierarchical, likely driven first by affective categorization, whereby pleasant/unpleasant and irritating sensory cues are extracted (seen at both physiological and semantic levels), followed by second-level analysis (seen at the semantic level) of features belonging to complementary perceptual dimensions.

The fact that pleasantness was the first salient dimension in the semantic and physiological spaces is clearly in agreement with previous studies of the organization of olfactory space. For instance, Schiffman^21–23^ found that the semantic distribution of odors, represented by a 2-dimensional space, tends to divide descriptors into two groups: on the one hand the more pleasant descriptors and on the other the unpleasant ones, suggesting that, in semantic description of odorants, the hedonic aspect is important for the perceptual organization of odorants. Likewise, Zarzo *et al*.^13,24,25^ explored the relationship between olfactory dimensions and semantic descriptors in several papers, and consistently found that odor semantic space was related to pleasantness. Moreover, edibility was also shown to be a salient dimension in the semantic space (although to a lesser degree than pleasantness): based on analysis of semantic space, several studies^9,13,26^ showed that edibility was one of the crucial sources of variability in the perceptual organization of smells. Likewise, Castro *et al*.^11^ found that, after pleasantness, the remaining dimensions identified appeared to correspond to cues for “palatability/unpalatability”. The present findings are also consistent with Haddad *et al*.’s study^1^, showing that the neural population response can predict both approach/avoidance behavior in animals (and pleasantness in humans), and the toxicity of an odorant molecule in animals (and edibility in humans).

One question that may be asked is whether the finding that pleasantness explain a large part of the variance in perceptual space of odors can be rooted in the neuro-anatomy of the olfactory system. Actually, in most mammals, the different olfactory stimulations from the environment can be processed by distinct neural route and this segregation of olfactory signals is observed already at the periphery of the system: the main olfactory epithelium is composed of several zones, activated differently according to the odorant molecules^27,28^, both as a function of the physicochemical properties of odorants but also according to the regionalized expression pattern of olfactory receptors^29^. These areas of the epithelium are connected to areas that are also distinct within the olfactory bulb. For example, the trace amine receptors are expressed in a dorsal zone of the olfactory epithelium and their activation specifically stimulates the glomeruli in the dorsal zone of the olfactory bulb^30^. In mice, stimulation of some of these receptors specifically trigger innate withdrawal behaviors^31^. In contrast, class II olfactory receptors are expressed within the ventral epithelium and activate the ventral zone of the olfactory bulb^32^ and are associated with learning of new odors. One assumption that may be raised - but that still need confirmation - is that like other mammals, the differential activation of certain receptor families involves the activation of distinct neuronal pathways and explains a part of the affective responses in human olfaction.

Analyzing autonomic nervous system responses to odors showed that, like for the semantic space, distances in the physiological spaces (especially latency, rise-time and amplitude of skin conductance response, and respiration) correlated significantly with distances in the pleasantness space of odors. These findings are clearly in line with previous studies suggesting that unpleasant odors are associated with increased heart rate and longer and stronger skin conductance responses compared to pleasant odors^20,33,34^. He *et al*.^35^ also showed that skin temperature in response to fish odor (considered unpleasant) was higher than that for a pleasant orange odor. As well as modulation of ANS responses by pleasantness, the present results further showed at the group level that variations in two trigeminal dimensions, irritation and pain, correlated with variations in ANS response. In the literature, little is known about how intranasal trigeminal sensations modulate ANS responses. One previous study showed that repeated exposure to a first trigeminal stimulation increased both the rated intensity and skin conductance for a subsequent odorant^36^. In a different context, another investigation revealed that trigeminal transcutaneous electrical stimulation (a technique that uses low-voltage electrical current for pain relief) reduced autonomic response in terms of heart rate variability and respiration rate under mental stress in healthy subjects^37^. However, to the best of our knowledge, no studies have focused on the influence of the intranasal trigeminal sensations conveyed by odorants on ANS responses.

These salient physiological responses to the irritating and painful aspect of odors likely reflect strong trigeminal system involvement in the “defensive” function of olfaction^38^. Interestingly, the same trigeminal aspects of odors (irritation and pain) were shown to be salient dimensions at a higher level of processing, in the semantic space of odors. The trigeminal system enables perception of chemosensory information from the environment and protects the organism from potential harm. For example, in the case of respiration, one can stop inhaling airborne irritants such as dust or chemicals^39^. Moreover, the trigeminal system enables us to detect the toxic molecule CO_2_, perceived as stinging and painful in the nasal mucosa when presented at high concentrations^40,41^. Another example of how the trigeminal system protects humans is the detection of propanethial-S-oxide, a lachrymatory gas produced by a series of enzymatic reactions when onions are cut^42^, which stimulates the ophthalmic branch of the fifth cranial nerve (trigeminal), causing the lachrymal glands to secrete tears which protect the eyes. The trigeminal system also allows us to identify appetitive sources such as coolness, conveyed by volatile molecules such as menthol^43^.

The neural route by which olfactory and trigeminal stimuli interact has been the topic of some investigations^44–48^. At the peripheral level, electrophysiological studies showed that interaction may take place in the mucosa whereby responses to olfactory stimuli are modulated by an axonal reflex of trigeminal afferents lying in the olfactory epithelium^49^. At the central level, it was shown that both the olfactory pathway and the trigeminal pathway converge on the same area within the thalamus (i.e. mediodorsal nucleus)^50^. Finally, brain imaging studies in humans revealed that olfactory and trigeminal stimuli activate separate but also common areas including the insula, cingulate cortex, and thalamus^51,52^.

The present findings revealed that the trigeminal system is a strong shaper of olfactory perception, acting at various processing levels, from non-verbal and basic physiological responses to more semantic and cognitively elaborate responses: the irritating and painful aspects of odors explain the main dimension of both the semantic and the physiological space of odors. To our knowledge, this study is one of the first to highlight such a finding, although previous studies suggested that sharpness and spiciness (qualities that may reflect trigeminal sensitivity)^22^ and pungency^53^ were principal dimensions in olfactory semantic space.

Beside these new findings, some of our methodological choices deserve discussion.

Firstly, one concern that may be raised regarding our method is why physiological signals were recorded in the first session whereas semantic data were collected in the second session. We chose to have two different sessions since the task involving physiological recording was too constraining for the semantic description task. Moreover, autonomic nervous system responses were always measured in the first session in order to avoid any familiarity effect in the physiological signal.

A second concern relates to the choice of the semantic description task in the second session. Previous research used different options to construct a semantic space of odors: odor pair similarity task, free description, or semantic description. Odor pair similarity tasks use a numeric scale to rate the similarity between a test odor and a series of reference odorants chosen as standards, or simply to rate similarity between odors without using a reference^24,54,55^. This method gives accurate results in studies with a small number of odorants, but is difficult and highly time-consuming when the odorant sample is large (which was the case in the present study). The second method (free description) consists in recording the words that participants freely recall when they smell the odorants. The main disadvantage of this method is the qualitative and quantitative heterogeneity of the data: odor identification is a very difficult task for lay participants, who are very often not able to make accurate description of odors. The third method (semantic description) consists in obtaining a semantic profile using a large set of predefined descriptors and, in some cases, numerically rating each attribute. We opted for this method, since it was frequently used in comparable studies, and because it generates data efficiently and rapidly^24,56^.

A third concern deals with the way these findings are interpreting in the light of the debate regarding the contribution of predetermined and acquired responses to odors. Defining the exact contribution of “innate” and “learning” mechanisms in shaping human perceptual and semantic spaces remains a difficult task in psychology and neuroscience. In olfaction studies, this issue has been approached by relating verbal features of odors (taken from a semantic space) to physical features of odorant molecules (taken from a physicochemical space). Psychophysical studies showed that some physicochemical parameters including molecular weight (MW), molecular complexity (MC) and sum of atomic van der Waals volumes (sV) explained partly the first components of the olfactory perceptual spacet^9,53,57,58^. In our study, to ask how these physicochemical parameters explained the organization of the semantic space, we extracted these three physicochemical properties from all 105 odorants using PubCHEM website and Dragon, a software application that enables calculation of thousands of molecular descriptors (Talete®). At the group level, correlation analyses revealed significant relationships between MC (r = −0.35, p < 0.001), MW (r = −0.43, p < 0.001) and sV (r = −0.47, p < 0.001) on the one hand and the first principal component of the semantic space on the other hand. When considering PC2, PC3 and PC4 of the semantic space no significant relationships were observed with these three physicochemical properties (except between MC and PC4, r = −0.35, p < 0.001). Thus, in line with previous studies^9,53,57,58^, predetermined responses to smells (through physicochemical treatment of odorants) partly organize the primary and main dimension of the semantic space of odors. These physicochemical properties of odorants molecules seem to play a very minor role in organizing the others dimensions of the semantic space leaving a broader role of learning and/or cultural mechanisms.

Another question that may be raised regarding our findings was the way we can interpret the sign of the correlation observed between the components of the semantic space and perceptual dimensions (often negative correlations; see for instance the correlation between pleasantness and the first principal component; Fig. 2a). The same question applies for the trigeminal dimensions (often positive correlations; except for coolness and warmth for some components; Fig. 2b). Actually, in the principal component analysis, individual vectors have arbitrary signs and there is no meaningful interpretation in terms of the data that the decomposition represents^59^. This sign depends among others factors on the computing analysis including the algorithm implementation that could differ from one method to another. Note that this arbitrary affectation of the sign for a given component is reflected in the individual analysis. Indeed, when considering the correlations between pleasantness and the first principal component of the semantic space, 13 individuals (among 22, 59%) had significant correlations. Among these 13 individuals, eight showed a negative correlation and five exhibited a positive correlation. Finally, results from the performed group analysis were not influenced by such sign affectation since the PCA results from this analysis were not a summation of all results from individual PCAs. Actually, the semantic matrix used to run the principal component analysis at the group level was an average of all semantic evaluations from all individuals.

To sum up, the present findings showed that the organization of both the semantic and the physiological space of odors is sustained by relevant perceptual dimensions such as pleasantness, enabling the organism to decide whether a sensory stimulus is attractive or repulsive. Importantly, our findings suggest that, in conjunction with pleasantness, trigeminal features of high biological importance are also involved in detecting relevant emotional stimuli, and modify the way humans categorize odorants. Our data also suggest a second-level analysis in which others dimensions are extracted (e.g. edibility, warmth). Beside their scientific impact, these findings also have methodological implications for future studies. Regardless of the techniques used to test the perception of odorants (experimental psychology, psychophysics, physiology, neurobiology), experiments should take account of the fact that intranasal trigeminal sensations can have an influence on the way mammals detect and represent smells in the brain.

## Methods

The study was conducted according to the Declaration of Helsinki and was approved by Lyon Sud-Est ethical review board. The experimental procedure was explained in detail to the participants and all gave written consent.

### Participants

Twenty-two participants (11 men and 11 women) between the ages of 19 and 46 years (mean ± SD, 32 ± 10 years) were recruited from the Lyon area in France and tested between April and June 2016. Six were smokers. All claimed normal sense of smell and were healthy (with no history of medical treatment, abnormal olfaction, neurological disease or injury or nasal insult).

### Odorants

A total of 105 odorants were used (molecules provided by Sigma-Aldrich). The odorants were presented in 15 mL flasks (opening diameter, 1.7 cm; height, 5.8 cm; filled with five ml of liquid) diluted in deodorized mineral oil to achieve an approximate gas-phase partial pressure of 1 Pa. Stimuli were absorbed on a scentless polypropylene fabric (3 × 7 cm; 3 M, Valley, NE, USA) to optimize evaporation and air/oil partitioning. Compound name, CID and volume/volume percentage dilution are given in Supplementary Table S1. Note that odorant selection was based on different criteria from physico-chemical characteristics, to olfactory and semantic. Here, the aim was to cover a large series of chemical families, such as alcohols, aldehydes, ketones, esters, pyrazines, furanones, acids, terpenes and sulfur compounds (with different molecular weights and odor qualities).

### Procedure

The experiment was divided in two sessions. The first consisted in recording physiological responses to odors, together with psychophysical ratings. The second consisted in psychophysical tests only. In both sessions, responses were computerized using custom-written software.

#### Session One

**Figure 5:**
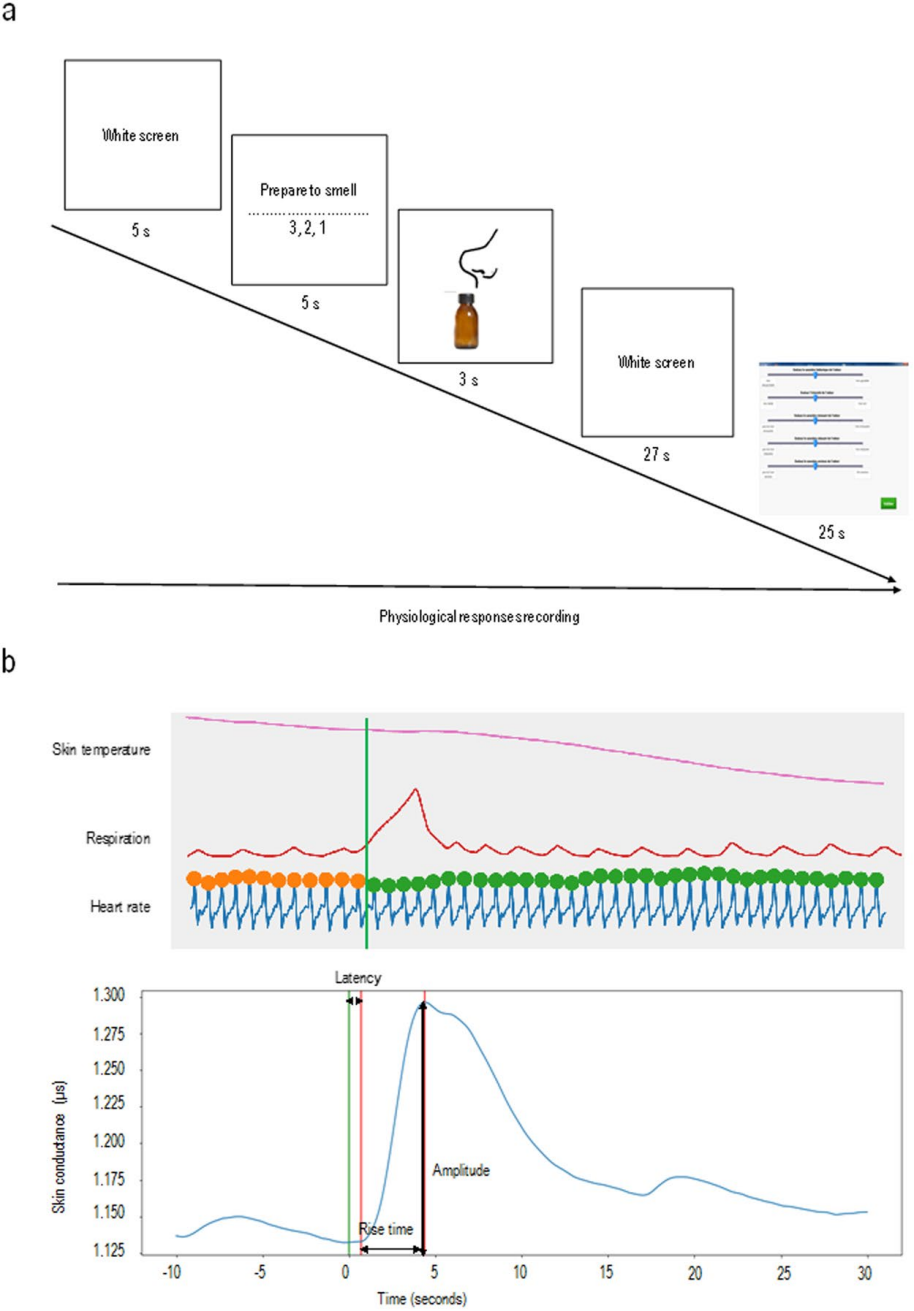
Experimental protocol. (**a**) Illustration of an odor trial in session one. (**b**) Example of physiological responses for one odor, in one participant.

Participants completed a demographic questionnaire, including age, gender, satiety level (scale from one for ‘not at all hungry’, to nine for ‘extremely hungry’), educational level (number of years of education), smoking habits and handedness. Participants were comfortably installed in a 7 × 7 × 4 m room, in a semi-reclined seated position. The room was aired before the experimental session. The experimenter fitted the ANS recording equipment to the participant. Once ANS measures had stabilized, odor trials were initiated. Participants were informed that they were going to smell several odors one after the other, and that each trial would begin with a white screen (for five seconds) followed by a written instruction, ‘Please prepare to smell’, followed by a countdown, ‘3, 2, 1’ (for five seconds), as shown in Fig. 5a.

They were instructed to sniff each flask for as long as it was presented, not to move and to focus their attention on the white screen in front of them. Odorant stimuli were presented by the experimenter about one cm below the subject’s nose, based on previous methodology^60^.Each stimulus was presented for three seconds. A rest period of 27 seconds followed odor presentation. Afterward, immediately after each odor trial, participants were instructed to rate both the pleasantness and intensity of the stimulus on a scale from one (not at all pleasant/intense) to nine (very pleasant/intense). They were also asked to rate the degree of relaxation, stress and anxiety induced by the odorants, on the same scale from one (not all relaxing, anxious, stressful) to nine (very relaxing, anxious, stressful) (data not analyzed in the current study). Participants could complete their ratings within a time window of 25 seconds. Before starting the experiment, they were trained with the experimental setting. All trials (105 odorants and four empty flasks containing only mineral oil) were divided in four sub-sessions or blocks (27 or 28 odorants/session), presented in random order. The duration of each block was around 30 minutes, followed by a five-minute pause. Total session duration was around three hours.

#### Session Two

In the second session, participants were told that they were going to smell a series of odorants, and their tasks were to rate them along different perceptual and semantic dimensions. The session comprised four sub-sessions, including one block of odorants (27 or 28 odorants/block) in each sub-session. For each trial, participants were asked to open the flask, and smell and describe the stimulus on a scale from low (0) to high (1), in terms of its quality, choosing from the following list of 24 predefined descriptors: floral rose, floral others, fruity citrus, fruity others, oily, vegetal, herbaceous, earthy, woody, smoky, cooked food, spicy, nutty, medicinal/minty/camphor, ethereal/disinfectant, alliaceous, chemical, fermentation, lactic, animal, human, decay, anisic and sweet/balsamic. These predefined quality descriptors were determined by first consulting several atlases widely used by perfumers to describe odorants^56,61–63^; in a second step, based on the study by Zarzo and Stanton^24^ where the authors described in detail various atlases and the similarities/dissimilarities between descriptors, we developed a list of 24 descriptors. These descriptors were common to all atlases, except for lactic and cooked food. They were carefully reviewed to avoid redundancies; thus, the chosen terms were general, and included several terms that corresponded to a specific semantic group: for example, the term “fruity” was chosen instead of “pear, banana, apple, pineapple, etc.”, and the term “earthy” included “moldy, humid and musty”. Importantly, any terms expressing intensity, pleasantness, emotions or trigeminal sensations, or other non-olfactory sensations (e.g., “clean”, “heavy”, “light”, “fresh”) were not included in the list. A pilot study (n = 13, six women) checked that the scale was easy to use, not tedious, and comprehensible.

Note that in the literature, there is no consensus as to the number of qualities to be used in a semantic scale, which depends on the experimental design. Nevertheless, Callegari, Rouault and Laffort^64^ determined that 25 well-chosen descriptors seemed sufficient to faithfully represent the perceptual olfactory space. Moreover, Jaeger and Ares^65^ concluded that whether few (10–17) or numerous terms (20–28) were used had limited impact on the characterizations elicited by some odorants; with longer lists, some qualities tended to be used less frequently. Based on these findings and after carefully choosing the descriptors, 24 descriptors were considered an adequate number. As stated above, the terms were ordered on a wheel scale based on a principal component analysis published by Zarzo and Stanton^24^, where descriptors were organized in terms of similarity in a semantic space constructed from the principal component analysis of several existing olfaction atlases. The wheel scale was inspired by previous studies in which aromatic wheels were developed to describe several foodstuffs such as wine^66,67^, beer^68^, crisps, cola or chocolate^69^.

During this second session, participants were also asked to rate all stimuli on a visual scale from 0 to one along the following dimensions: pleasantness, intensity, familiarity, edibility, coolness, warmth, pain and irritation. Finally, participants were asked to rate each stimulus for the following 6 basic emotional states (not analyzed in the present study): happiness, sadness, fear, anger, disgust, and surprise; neutral state and pleasure were also added. All scales and terms were presented in French. Participants carried out the tasks at their own pace, at a mean one stimulus/min. As in session one, the duration of session two was around three hours. It should be mentioned here that we quantified the fatigue evoked by the task by asking participants to rate their tiredness after each block using a scale from 1 (not at all tired) to 9 (very tired). No significant difference in fatigue was noted across blocks (F(3,57) = 1.833, p > 0.050).

It is important to mention that participants rated the hedonic value and intensity of the molecules in both sessions, so as to test the reproducibility of the results from one session to the other.

### Physiological recordings

Different psychophysiological parameters were simultaneously and continuously recorded and displayed during the experiment: finger pulse frequency, skin conductance, skin temperature and abdominal respiration (Fig. 5b).

Skin conductance amplitude (in microsiemens: μS) was recorded by two circular Ag/AgCl electrodes (diameter one cm) placed on the third phalanx of the forefinger and of the middle finger of the non-dominant hand. We first applied a Gaussian filter to the data, using 1 Hz as cutting frequency, and then reduced them to latency (ms), rise time (ms) and amplitude (μS).

Finger pulse frequency was measured using a photoplethysmographic probe (3.2 cm/1.8 cm, LED photodetector) placed on the thumb of the non-dominant hand. Data were reduced to pulse rate, in beats per minute (bpm).

Skin surface temperature was measured using a small ceramic-encapsulated metal-oxide semiconductor (9.5 mm length, two mm diameter). The thermistor, designed to operate from 0 °C to 50 °C, was placed directly on the first phalanx of the fourth finger. Data were reduced to mean skin temperature.

Changes in abdominal circumference associated with respiration were measured using a respiratory belt transducer (100 cm rest length, ten cm maximum elongation, 3.5 cm width), responding linearly to changes in length. Data were reduced to respiratory frequency, in cycles per minute (cpm).

All parameters were analyzed in the 30-s window after stimulus onset (using the ten seconds before odor presentation as baseline). Abdominal respiration, skin temperature and finger pulse frequency were normalized by subtracting baseline values from the values in the 30-s window after stimulus onset. Data were sampled and recorded at 256 Hz, then converted and amplified via an 8-channel PROCOMP+ system (Thought Technology, Montreal, Canada) and displayed, stored, reduced and analyzed off-line.

### Data analysis

As a first step, a matrix was constructed with the 105 odorants as rows and 38 variables per odorant as columns: the 24 semantic descriptors, eight olfactory dimensions and six physiological responses. Each cell value represented the average value (across subjects) for each of the 38 variables. For physiological response analysis, three participants were excluded due to technical problems with data recording. For perceptual data, some participants did not perceive certain odorants, and n-values varied from 17 to 22 (and 15 for ethyl acetate).

To assess the reproducibility of results between sessions, the pleasantness and intensity ratings obtained in session one and session two were compared, by a Pearson correlation between the two sessions for the two variables.

Next, to build a semantic space of odors, we conducted a principal component analysis (PCA, in Tanagra®), using the 24 semantic descriptors of the 105 odorants (and excluding the eight dimensions of intensity, pleasantness, familiarity, edibility, irritation, pain, warmth and coolness). The same analysis was made for the physiological space; here, the data consisted in a vector of six physiological parameters. In both cases, after extracting the principal components, a parallel analysis was followed by a bootstrap analysis to determine the number of significant principal components. All cases used 200 replications and a confidence level of 0.95 and 0.90 were used for the parallel and bootstrap analysis respectively.

To understand the meaning of each significant principal component of the two PCAs (semantic space and physiological space), correlation coefficients were calculated between the significant principal components and the attributes of interest (perceptual attributes: pleasantness, intensity, edibility and familiarity; trigeminal attributes: irritation, pain, warmth and coolness). When a significant effect was observed, the analysis was followed by a false discovery rate (FDR) correction for multiple statistical comparisons.

Finally, a similarity analysis was used to assess how semantic (and physiological) responses to the 105 odorants related to each of the eight perceptual dimensions. Here, a semantic (and physiological) space was constructed by extracting the significant principal components provided by the PCAs. The PCA showed four significant components for the semantic space (eigenvalues: PC1 = 4.54, PC2 = 2.57, PC3 = 2.32, PC4 = 2.03) and one component for the physiological space (eigenvalue: PC1 = 2.17). The similarity in semantic (and physiological) patterns between odorants was represented by a matrix comprising the 105 experimental odorant conditions. In this matrix, each cell value represented the semantic (or physiological) distance (or degree of dissimilarity) between pairs of stimuli, measured as a Euclidean distance. To measure similarity in each of perceptual dimensions, a one-dimensional space for intensity, pleasantness, familiarity, edibility, irritation, pain, warmth and coolness ratings was constructed, resulting in a distance matrix or space between the 105 odorants for each dimension. A Mantel test^47^ was used to compare the semantic (and physiological) matrix on the one hand, and each of the 8 perceptual matrices; this test included a permutation test (1,000 permutations) to assess the significance of the comparisons. A distance similarity (DS) index, corresponding to one minus the Pearson correlation coefficient, was computed for each comparison. Since multiple testing was performed, p-values were adjusted using an FDR correction.

## Electronic supplementary material

Supplementary Table S1
