# Supplementary material for: Pleasantness and trigeminal sensations as salient dimensions in organizing the semantic and physiological spaces of odors
Source: Sci Rep. 2018 May 31;8:8444. doi: 10.1038/s41598-018-26510-5 (PMC5981304; doi:10.1038/s41598-018-26510-5)
Supplement: Supplementary file 1 — Supplementary Table S1 [file 41598_2018_26510_MOESM1_ESM.doc]

**Pleasantness and trigeminal sensations as salient dimensions in organizing the semantic and physiological spaces of odors**

Licon C. C., Manesse C., Dantec M., Fournel A., Bensafi M.

Supplementary Table S1. Odorants used in the Experiment and their CID and percentage dilution (vol/vol) in mineral oil

| **Compound**  **name** | **CID** | **Dilution % (vol/vol)** |
| --- | --- | --- |
| (-)-fenchone | 2794921 | 0.67 |
| (-)-terpinen-4-ol | 5325830 | 5 |
| (+)-camphor | 2537 | 0.46 |
| (+)-fenchone | 1201521 | 0.68 |
| (+)-linalool | 6549 | 2.17 |
| 1-butanol | 263 | 0.05 |
| 1-decanol | 8174 | 43.31 |
| 1-heptanol | 8129 | 1.26 |
| 1-octen-3-ol | 18827 | 0.56 |
| 1-octen-3-one | 61346 | 0.27 |
| 1-propanol | 1031 | 0.05 |
| 2,3-butanedione | 650 | 0.00298 |
| 2,3-dimethylpyrazine | 22201 | 0.06 |
| 2-butanone | 6569 | 0.5 |
| 2-ethyl-3,5-dimethylpyrazine | 26334 | 0.2 |
| 2-heptanone | 8051 | 0.07 |
| 2-methyl butyric acid | 8314 | 0.38 |
| 2-methyl-butanal | 7284 | 0.00042 |
| 2-methyl-propanal | 6561 | 0.00010 |
| 2-nonanone | 13187 | 1 |
| 2-phenylethanol | 6054 | 2.66 |
| 3-hexanol | 12178 | 0.07 |
| 3-methyl-1-butanol | 31260 | 0.09 |
| 3-methyl-butanal | 11552 | 0.00414 |
| 3-methyl-butanoic acid | 10430 | 0.48 |
| 3MHA  (3-methylhex-2-enoic acid) | 6443739 | 4 |
| 4-ethyloctanoic acid | 61840 | pure |
| 4-methylphenol  (p-cresol) | 2879 | 0.5 |
| Acetic acid | 176 | 0.01 |
| Acetophenone | 7410 | 0.57 |
| Allyl caproate | 31266 | 0.56 |
| Androstadienone  (androsta-4,16-dien-3-one) | 92979 | 0.575 |
| Androstenol | 101989 | 2.5 |
| Androstenone | 6852393 | 0.75 |
| **Compound**  **name** | **CID** | **Dilution % (vol/vol)** |
| Benzaldehyde | 240 | 2 |
| Benzyl acetate | 8785 | 1.55 |
| Butanoic acid | 264 | 0.11 |
| Butyl acetate | 31272 | 0.02 |
| Butyl butyrate | 7983 | 0.18 |
| Butyl propionate | 11529 | 1 |
| Cineole | 2758 | 1 |
| Cis-3-hexenol | 5281167 | 0.24 |
| Cis-3-hexenylacetate | 15574 | 0.25 |
| Citral | 638011 | 3.62 |
| Citronellal | 7794 | 1.43 |
| Citronellol | 8842 | 17.85 |
| D-camphor | 159055 | 0.46 |
| D-carvone | 7439 | 1.93 |
| Decanal | 8175 | 3.53 |
| Decanoic acid | 2969 | 11.4 |
| Dimetylsulphide | 1068 | 0.00010 |
| D-limonene | 440917 | 0.16 |
| Dodecanal | 8194 | 12.59 |
| Ethyl acetate | 8857 | 0.02 |
| Ethyl butanoate | 7762 | 0.05 |
| Ethyl hexanoate | 31265 | 0.19 |
| Ethyl octanoate | 7799 | 1.71 |
| Ethyl phenyl acetate | 7590 | 5.11 |
| Ethyl salicylate | 8365 | 5.49 |
| Ethyl vanillin | 8467 | 26% |
| Ethyl-2-methyl-propanoate | 7342 | 0.05 |
| Ethyl-3-methyl-butanoate | 7945 | 0.04 |
| Eugenol | 3314 | 13.45 |
| Furaneol | 19309 | 7.37 |
| Geraniol | 637566 | 11.29 |
| Guaiacol | 460 | 2.09 |
| Heptanal | 8130 | 0.08 |
| Hexanal | 6184 | 0.1 |
| Hexanoic acid | 8892 | 2 |
| **Compound**  **name** | **CID** | **Dilution % (vol/vol)** |
| HMHA  (3-hydroxy-3-methylhexanoic acid) | 16666688 | 0.1 |
| Indole | 798 | 2 |
| Iso amyl phenyl acetate | 7600 | 80.6 |
| Isoamyl acetate | 31276 | 0.03 |
| Isobutyric acid | 6590 | 0.1 |
| L-carvone | 439570 | 1.93 |
| Menthol | 16666 | 11.17 |
| Methional | 18635 | 0.05 |
| Methyl anthranilate | 8635 | 8 |
| Methyl salicylate | 4133 | 7.26 |
| MSH  (3-methyl-3-sulfanylhexan-1-ol) | 10130039 | 0.00299 |
| Myrcene | 31253 | 0.15 |
| Nonanal | 31289 | 0.9 |
| Pentanal | 8063 | 0.01 |
| Pentanol | 22386 | 0.05 |
| Phenyl ether | 7583 | 13.58 |
| Phenylacetaldehyde | 998 | 0.58 |
| Phenylethyl acetate | 7654 | 5.48 |
| Propanoic acid | 1032 | 0.04 |
| Propyl acetate | 7997 | 0.2 |
| Propyl butyrate | 7770 | 0.05 |
| Propyl propionate | 7803 | 0.05 |
| Pyridine | 1049 | 0.01 |
| S-(-)-limonene | 439250 | 1 |
| Thioglycolic acid | 1133 | 1.55 |
| Thymol | 6989 | 18.6 |
| Trans-2-hexenal | 5281168 | 0.05 |
| Trans-2-hexenylacetate | 17243 | 0.16 |
| Trans-anethole | 637563 | 4.17 |
| α-ionone | 5282108 | 20.5 |
| α -pinene | 440968 | 1 |
| α-santalol | 5368797 | pure |
| α-terpinene | 7462 | 1.5 |
| β-caryophyllene | 5281515 | 33.64 |
| β -ionone | 638014 | 7.27 |
| γ-Decanolactone | 12813 | 10 |
